# Flotillin‐1 is a prognostic biomarker for glioblastoma and promotes cancer development through enhancing invasion and altering tumour microenvironment

**DOI:** 10.1111/jcmm.17660

**Published:** 2023-01-17

**Authors:** Ran Wang, Zhikang Chen, Yi Zhang, Shihan Xiao, Wuming Zhang, Xianqin Hu, Qun Xiao, Qing Liu, Xiangyu Wang

**Affiliations:** ^1^ Department of Colorectal and Anus Surgery, Xiangya Hospital Central South University Changsha Hunan China; ^2^ The Hunan Provincial Key Lab of Precision Diagnosis and Treatment for Gastrointestinal Tumor, Xiangya Hospital Central South University Changsha Hunan China; ^3^ Department of Neurosurgery, Dengzhou People's Hospital Dengzhou Henan China; ^4^ Department of Neurosurgery, Xiangya Hospital Central South University Changsha Hunan China

**Keywords:** flotillin‐1, glioblastoma, invasiveness, prognostic biomarker, tumour microenvironment

## Abstract

Flotillin‐1(FLOT1) has long been recognized as a tumour‐promoting gene in several types of cancer. However, the expression and function of FLOT1 in glioblastomas (GBM) has not been elucidated. Here, in this study, we find that the expression level of FLOT1 in GBM tissue was much higher than that in normal brain, and the expression was even higher in the more aggressive subtypes and IDH status of glioma. Kaplan–Meier survival revealed that high FLOT1 expression is closely associated with poor outcome in GBM patients. FLOT1 knockdown markedly reduced the proliferation, migration and invasiveness of GBM cells, while FLOT1 overexpression significantly increases GBM cell proliferation, migration and invasiveness. Mechanistically, FLOT1 expression may play a potential role in the microenvironment of GBM. Therefore, FLOT1 promotes GBM proliferation and invasion in vitro and in vivo and may serve as a biomarker of prognosis and therapeutic potential in the fight against GBM.

## 1 INTRODUCTION

Glioblastomas (GBM) is the most common and aggressive primary brain tumour, which are thought to originate from neuroglial stem or progenitor cells.^1^ The intra‐ and intertumoral heterogeneity and blood–brain barrier are thought to be the obstacles for effective treatment.^2^, ^3^ In recent years, the redundant signalling pathways and immunosuppressive microenvironment also contribute to GBM treatment resistance.^4^ Unfortunately, the currently applied therapeutic strategies, including advanced neurosurgery, radiotherapy and chemotherapy, are far from satisfactory.^5^ Therefore, it is high time to investigate novel biomarkers and therapeutic targets to improve the treatment of gliomas.

Flotillin‐1 (FLOT1), member of the flotillin family (also known as the reggie family), is a hallmark of lipid rafts, who helps form a platform on plasma membrane or intracellular organelles^6^ for various molecules to conduct their functions, including TNFα and TNFR,^7^ Src tyrosine kinases (Src, Fyn and Lck) and small GTPases.^8^, ^9^ FLOT1 has a great impact on numerous biological processes, including endocytosis, adhesion, actin cytoskeleton reorganization and cell‐signalling events.^10^, ^11^, ^12^, ^13^ Recently, several cancer types are reported to be associated with FLOT1, including breast, renal, oesophageal, colorectal, prostate and lung carcinoma.^14^, ^15^, ^16^ However, the function and expression of FLOT1 in GBM progression and the tumour microenvironment have not been investigated.

In this study, we found that FLOT1 was upregulated in both GBM tissue and cells. GBM cells with FLOT1 knockdown displayed marked reductions in proliferation, migration and invasiveness. While overexpression of FLOT1 increased the proliferation, migration and invasiveness of glioma cells via MAPK signalling. Additionally, FLOT1 expression was associated with stromal and immune cell infiltration in GBM.

## 2 MATERIALS AND METHODS

### 2.1 Cell lines and culture

We bought human glioma cell lines (U87, U251) from ATCC (Manassas, Virginia, USA), and the cells were cultured in foetal bovine serum supplemented DMEM (10% FBS + DMEM) in cell incubator (37°C, 5% CO_2_).

### 2.2 Lentiviral transfection and Flot1 shRNA gene silencing

The lentiviral vector LV19‐Flot1 containing the Flot1 overexpression gene and puromycin resistance gene, Flot1 shRNA(GCAGAGAAGTCCCAACTAATT), contains the puromycin resistance gene, and the corresponding controls were transfected into U87 and U251. Transfected cells were cultured with DMEM containing puromycin(2 μg/ml) for 14 days. Then, we used the transfected cells to extract protein.

### 2.3 Western blotting

Protein samples mixed with 5Xloading buffer were boiled at 100°C for 5 min. Then, cooled down the samples and subjected them into sodium dodecyl sulfate–polyacrylamide gel, electrophoresed and transferred the sample to PVDF membrane. Then, blocked the membrane with 5% skimmed milk for 1 h, and then incubated the membrane with primary antibodies, anti‐Flot1 (1:1000, Proteintech, Rosemont, IL, USA), anti‐p‐ERK1/2 (Thr202/Tyr204) (1:1000, Proteintech) at 4°C overnight. Washed the membrane, incubated it into secondary antibodies, and washed it again 3 times. At last, detected the signals with ChemiDoc (Bio‐Rad).

### 2.4 Cell proliferation assay

Inoculated cells (2 × 10^3^) into 96‐well plates one night before. Add 10 μl MTT (3‐ (4,5)‐dimethylthiahiazo (‐z‐y1)‐3,5‐di‐phenytetrazoliumromide) solution into each well and incubated in the cell incubator, 4 h later add 100 μl DMSO into each well after MTT solution was removed. The absorbance of each well was measured at 570 and 630 nm with a Spectra Max M2 Microplate Reader (Molecular Devices).

### 2.5 Cell wound healing assay

Inoculated cells (5 × 10^5^) into a 12‐well plate. Scratched the plate using 10 μl pipettes after cell adherence, washed the plate with PBS buffer, and then cultured cells in DMEM without serum. Observed wound closure at 0 and 24 h. Image J (1.4.3.67) was used to evaluate cell migration ability after the images were taken in microscopic fields three times randomly (× horizon200) at both time points.

### 2.6 Transwell invasion assay

Suspended U87 and U251 Cells (5 × 10^4^) were placed in the upper chamber of 8‐μm‐pore Transwells (BD Biosciences) precoated with Matrigel, and the lower chamber was filled with DMEM with 20% FBS to attract cells. Culture cells in cell incubator for 2 days. The 8‐μm hole was stained with 0.5% crystal violet for 30 min after fixation (4% paraformaldehyde), and three microscopic fields (× horizon 200) were selected randomly under the microscope and then count cells.

### 2.7 Xenograft mouse model

Eight NOD‐SCID male mice of 4 weeks old were divided into 2 groups randomly. One group was injected with U87 FLOT1‐OE cells (1 × 10^6^) on the back under skin, and the other group were injected with control cells. All the mice were monitored, and tumour size was measured every 6 days. We used the formula V = (Length × Width2) to calculate tumour volume.

### 2.8 Datasets

We obtained clinical information and RNA‐seq data for 143 GBM samples from TCGA cohort. By searching the Genotype‐Tissue Expression (GTEx) cohort, we got RNA‐seq data for 1151 normal brain samples. Both datasets were accessed through the Xena browser (http://xena.ucsc.edu/). RNA‐seq data were converted to transcripts per kilobase of exon model per million mapped reads and transformed by log2(X + 1).

By searching the Chinese Glioma Genome Atlas in GlioVis (http://gliovis. Bioinfo.cnio.es/), we got clinical information and normalized RNA‐seq data for 133 GBM patient samples.^12^ Samples with complete prognostic information were included in this study, and we have summarized the basic clinical characteristics of all samples in Table S1. In addition, we adopted immunohistochemistry images of FLOT1 protein expression in normal brain and GBM patients' tissue from The Human Protein Atlas.^17^

### 2.9 Gene enrichment analysis

In this study, FLOT1‐related genes were identified in the TCGA and CGGA cohorts, respectively, based on Spearman correlation analysis with a threshold of |*R*| >0.6 and *p* < 0.05. Based on FLOT1‐related genes, the R package ‘cluster Profiler’ was used to perform Kyoto encyclopaedia of genes and genomes (KEGG) pathway analysis and Gene ontology (GO) enrichment analyses.^18^ *p* < 0.05 was considered statistically significant.

### 2.10 Immune infiltration analysis

Using the methods from Yoshihara et al.,^19^ the ESTIMATE algorithm was used to estimate ImmuneScore, StromalScore and ESTIMATEScore in GBM samples. We did single‐sample gene set analysis to quantify the relative abundance of 28 previously reported immune cells.^20^ In addition, by using TIMER tool, we explored the relationship exists between FLOT1 expression and the abundance of six major immune cell infiltration (https://cistrome.shinyapps.io/timer/).^21^

### 2.11 Statistical analysis

The Student's *t*‐test was used to analyse the two groups of data. The statistical significance levels in this study are as follows: * < 0.05, ** < 0.01, *** < 0.001 and**** < 0.0001. The experiments were repeated three times.

## 3 RESULTS

### 3.1 FLOT1 is highly expressed in GBM tissue and associated with clinicopathology characters and patients’ outcome

**FIGURE 1:**
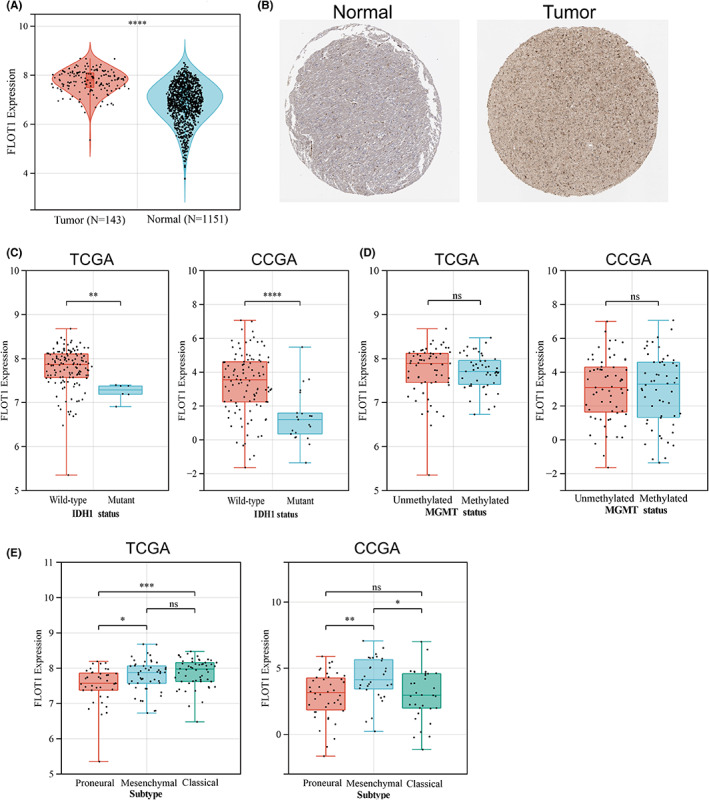
Expression level of FLOT1 in GBM and its subtypes. (A) Differential expression of FLOT1 between GBM and normal brain tissue. (B) IHC staining of FLOT1 in GBM and normal brain tissue. (C) FLOT1 expression in IDH1 wildtype and IDH1 mutant samples based on TCGA and CGGA database. (D) The expression of FLOT1 in MGMT‐methylated and MGMT‐unmethylated samples based on TCGA and CGGA database. (E) The expression of FLOT1 in different GBM subtypes based on TCGA and CGGA datasets.

Given that FLOT1 was overexpressed in several types of cancer, we investigated the expression of FLOT1 in human GBM samples and normal brain tissue by querying TCGA and GTEx databases. We found that the expression level in GBM tissue is much higher than that in normal brain (*p* < 0.0001) (Figure 1A). Meanwhile, in contrast to normal brain tissue, GBM samples exhibited an increased staining intensity (Figure 1B, Figure S3A,B).

As is well known, IDH1 and MGMT status affected GBM patients' outcome. According to the data in CGGA and TCGA databases, FLOT1 was highly expressed in IDH‐WT samples (Figure 1C), but no significant difference was found between methylated and unmethylated MGMT samples (Figure 1D). Furthermore, we investigated the expression of FLOT1 in different GBM transcriptional subtypes. Although there was a distinct trend between these three subtypes according to the TCGA and CGGA databases, we found that FLOT1 was highly expressed in the mesenchymal subtype (Figure 1E).

**FIGURE 2:**
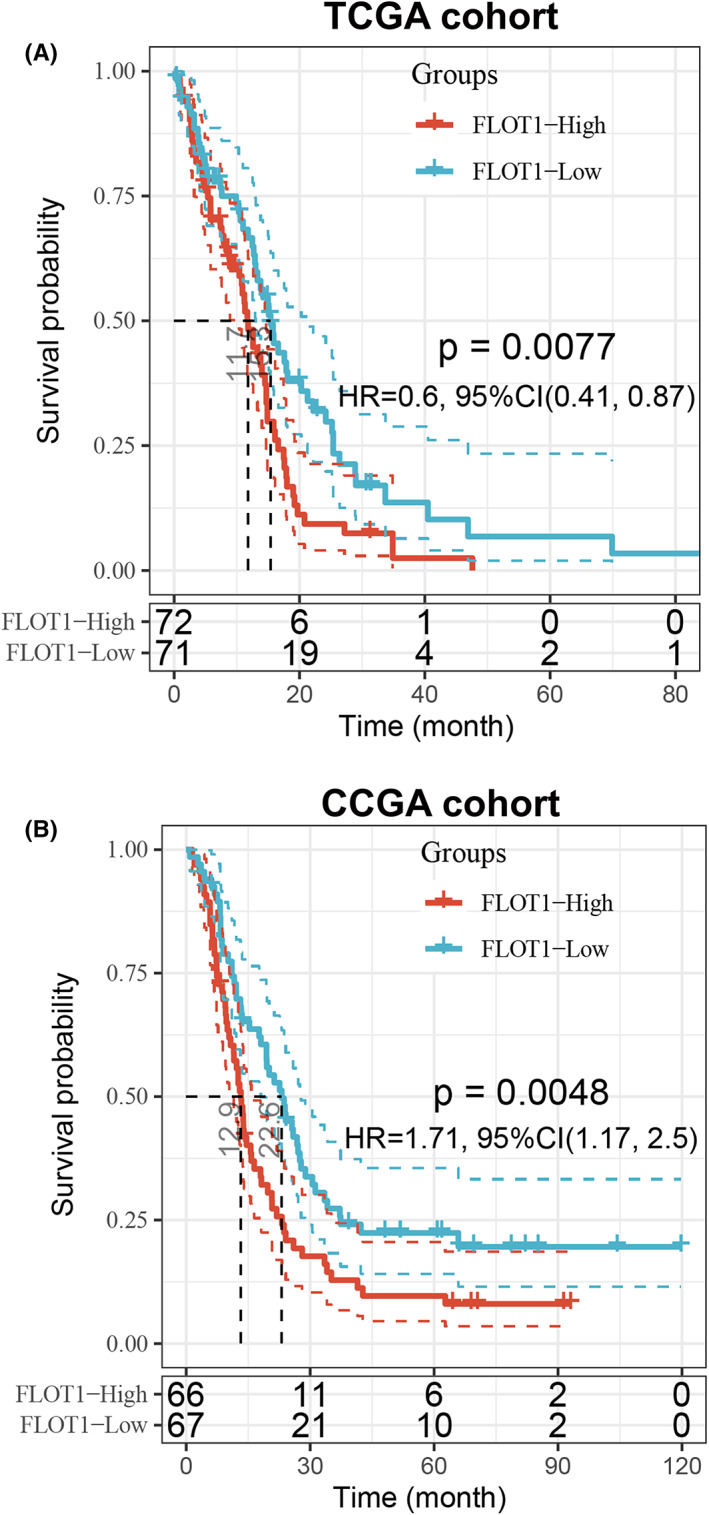
FLOT1 could be a prognostic factor for GBM patients. (A) Kaplan–Meier survival analysis shows higher FLOT1 expression predicts a poorer prognosis for GBM patients based on the TCGA database. (B) Kaplan–Meier survival analysis shows higher FLOT1 expression predicts a poorer prognosis for GBM patients based on the CGGA database.

To further study the effect of FLOT1 on GBM, we also did Kaplan–Meier survival analysis, and the results showed that patients with high FLOT1 expression showed shorter overall survival time (Figure 2A–B). Taken together, GBM tissue showed higher level of FLOT1, and patients with high FLOT1 expression came out with poor prognosis.

### 3.2 FLOT1 promotes proliferation, migration and invasion in glioma cells

**FIGURE 3:**
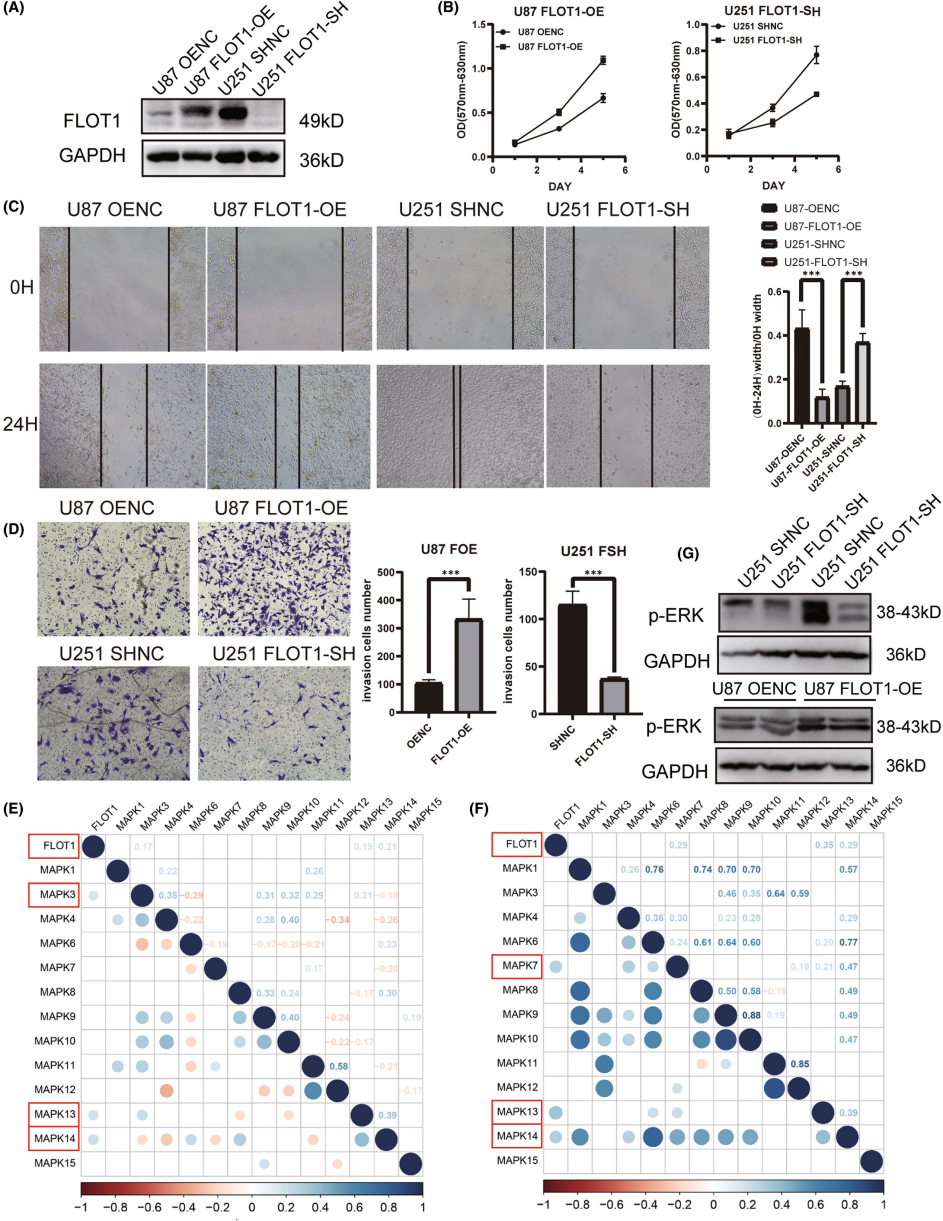
FLOT1 is associated with GBM proliferation, migration, invasion and p‐ERK level in vitro. (A) Western blot shows the FLOT1 expression level in FLOT1 overexpressed U87 cells and FLOT1 knockdown U251 cells. (B) MTT assay reveals the proliferation ability of GBM cells, which overexpressed FLOT1 or knocked down FLOT1. (C) The wound healing assay demonstrates the migration ability of GBM cells, which overexpressed FLOT1 or knocked down FLOT1. (D) Transwell assay shows the invasive ability of GBM cells in which FLOT1 was overexpressed or knocked down. (E, F) The relationship between FLOT1 and MAPK signalling according to the TCGA and CGGA database. (G) Western blot shows the p‐ERK level difference between control and FLOT1 overexpressed U87 cells. All in vitro experiments were performed in triplicate and at least three times.

To investigate the role of FLOT1 in GBM, U87 and U251 cells were transfected with FLOT1‐OE and FLOT1‐SH lentivirus, respectively, to overexpress and knock down the expression level of FLOT1. Cells transfected with corresponding empty lentiviral vectors were used as negative controls. We confirmed the transfection effectiveness using western blotting (Figure 3A). The MTT assay, cell wound healing assay and transwell invasion assay were used to compare cell proliferation rates, migration and invasion ability among FLOT1‐OE, FLOT1‐SH and their control cells. Cell proliferation, migration and invasion abilities of U87 cells were significantly enhanced when FLOT1 was overexpressed. Cell proliferation, migration and invasion rates were inhibited by knocking down FLOT1 in U251 cells (Figure 3B–D). FLOT1 has been reported to be a very important regulator of classical MAP kinase signalling. Monia Amaddii and colleague^22^ demonstrated that, at the late phase of growth factor stimulation, FLOT1 binds to CRAF, MEK1 and ERK, which are core components of three tiers of classical MAPK signalling, and that knockdown of FLOT1 results in a direct inactivation of ERK1/2. Therefore, we explored the correlation between FLOT1 and MAPK signalling in GBM using TCGA and CCGA databases(Figure 3E–F). We also tested the expression levels of p‐ERK1/2, one of the most important active components in the MAPK transduction system, in U87 FLOT1‐OE and control cells. The results showed that FLOT1 overexpression significantly upregulated p‐ERK1/2 (Figure 3G).

### 3.3 FLOT1 promotes GBM tumour growth in xenograft mouse model

**FIGURE 4:**
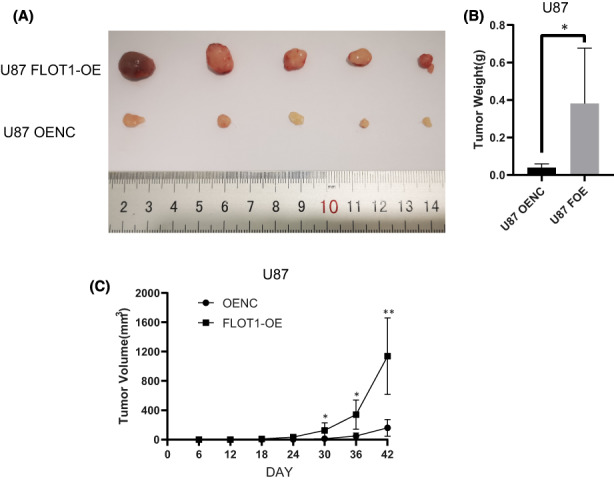
FLOT1 promotes GBM tumour growth in xenograft mouse model. (A) Picture of tumours from FLOT1‐OE and control group 42 days after cells were inoculated. (B) Tumours were weighed. (C) Tumour sizes were monitored every 6 days.

To further investigate the role of FLOT1 in GBM development, we inoculated FLOT1‐OE and control U87 cells into NOD‐SCID mice on the back under skin. Mice were sacrificed, and tumours were collected and weighed 40 days later. We found that the tumours from FLOT1‐OE group were larger in size and weight than that in control group, which indicated that FLOT1 overexpression dramatically increased GBM xenograft tumour development (Figure 4A–C).

### 3.4 Functional enrichment of FLOT1 in GBM

**FIGURE 5:**
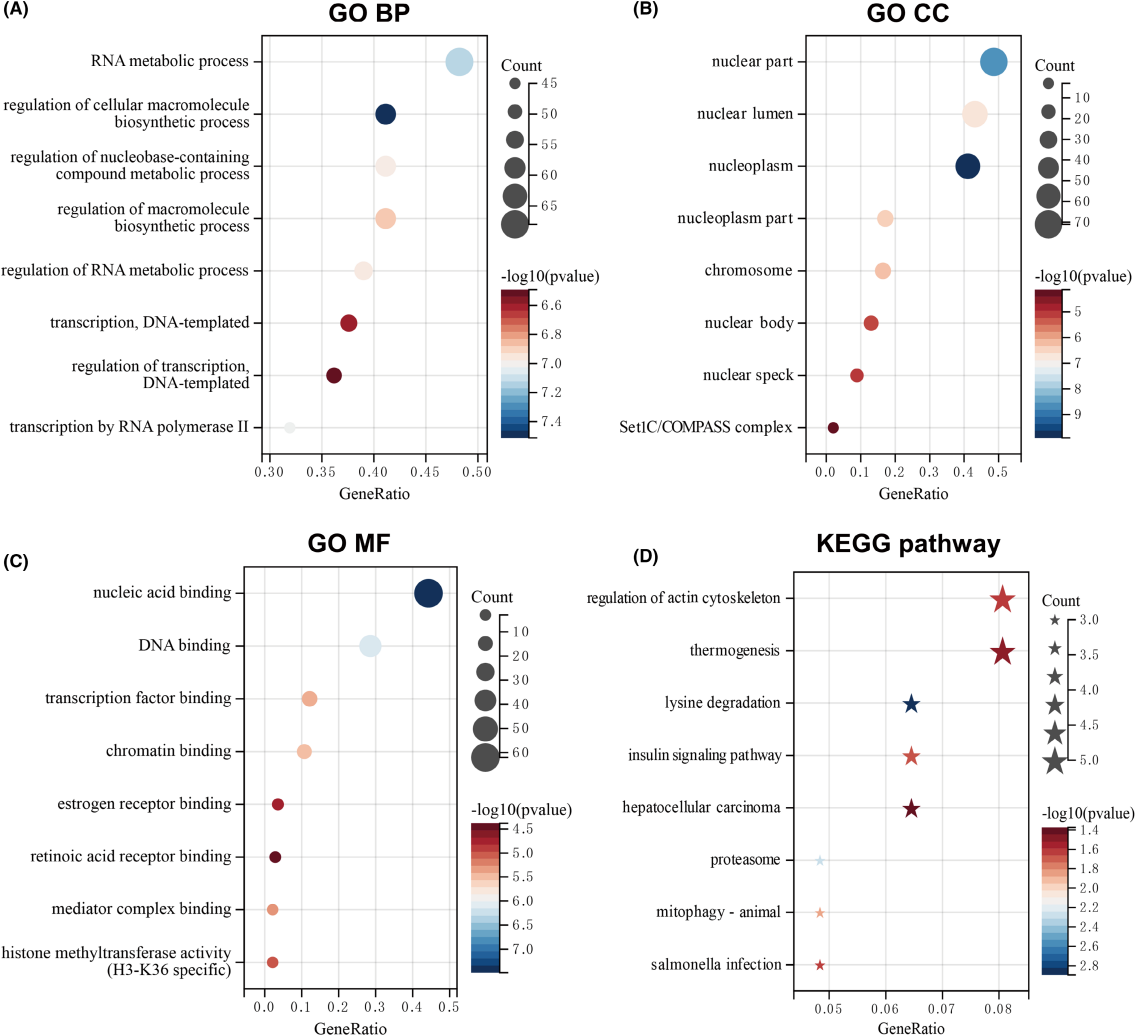
Functional enrichment analysis of FLOT1 in GBM patients (A) Top eight biological process (BP) terms in GO enrichment based on TCGA database. (B) Top eight cellular component (CC) terms of FLOT1 in the TCGA database. (C) Top eight molecular function (MF) terms of GO enrichment based on TCGA database. (D) KEGG pathways were analysed based on TCGA datasets, and the top 8 terms were listed.

To investigate the mechanism of FLOT1 in GBM, we first identified FLOT1‐related genes in TCGA cohorts, using Spearman correlation analysis with a threshold of |*R|* >0.6 and *p* < 0.05. The results showed that 180 genes were related to TCGA (Table S2). Next, we did GO enrichment and KEGG pathway analyses (Tables S3 and S4). GO analysis identified 487 terms of biological process, 73 terms of cellular component and 105 terms of molecular function from the TCGA database. The top eight terms of the biological processes were mainly enriched in the functions of RNA metabolic process and DNA transcriptional processes (Figure 5A). The top eight cellular component terms were significantly associated with the nucleus (Figure 5B). The top eight molecular functions were mainly enriched in nucleic acid binding, DNA binding and transcription factor binding (Figure 5C). Meanwhile, KEGG pathway analysis revealed that FLOT1 correlated with the regulation of the actin cytoskeleton, thermogenesis, lysine degradation and insulin signalling pathway (Figure 5D). GO enrichment and KEGG pathway analyses were also performed based on CGGA database (Figure S1A–D, Tables S5 and S6). These results indicated that FLOT1 might be associated with transcriptional regulation and cell morphology.

### 3.5 FLOT1 expression is associated with stromal and immune cell infiltration in GBM

**FIGURE 6:**
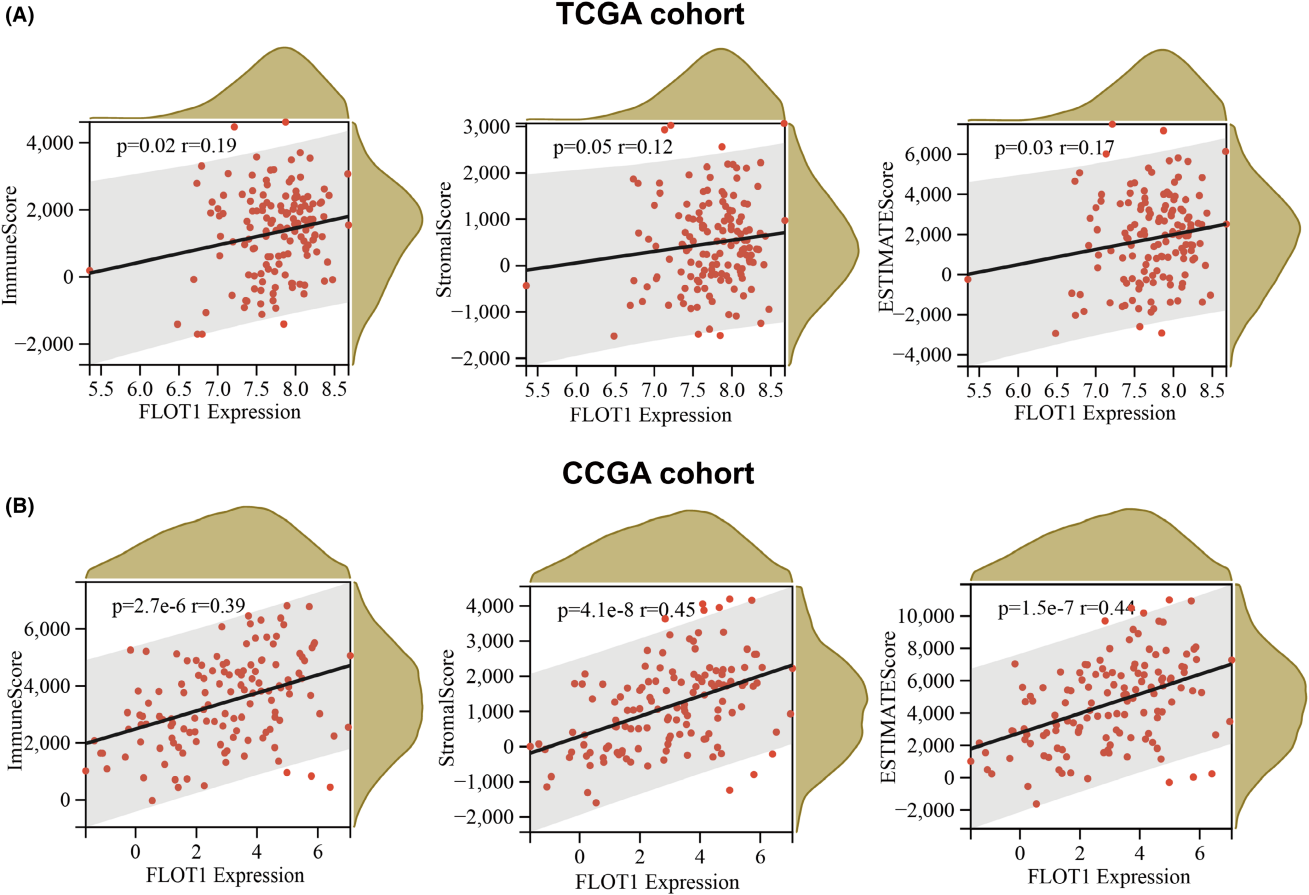
Positive correlation between the FLOT1 expression and the immune score in GBM patients. (A) The correlation between the expression of FLOT1 and ImmuneScore, StromalScore and ESTIMATEScore basing on the TCGA database. (B) The correlation between FLOT1 expression and ImmuneScore, StromalScore and ESTIMATEScore basing on the CGGA database.

**FIGURE 7:**
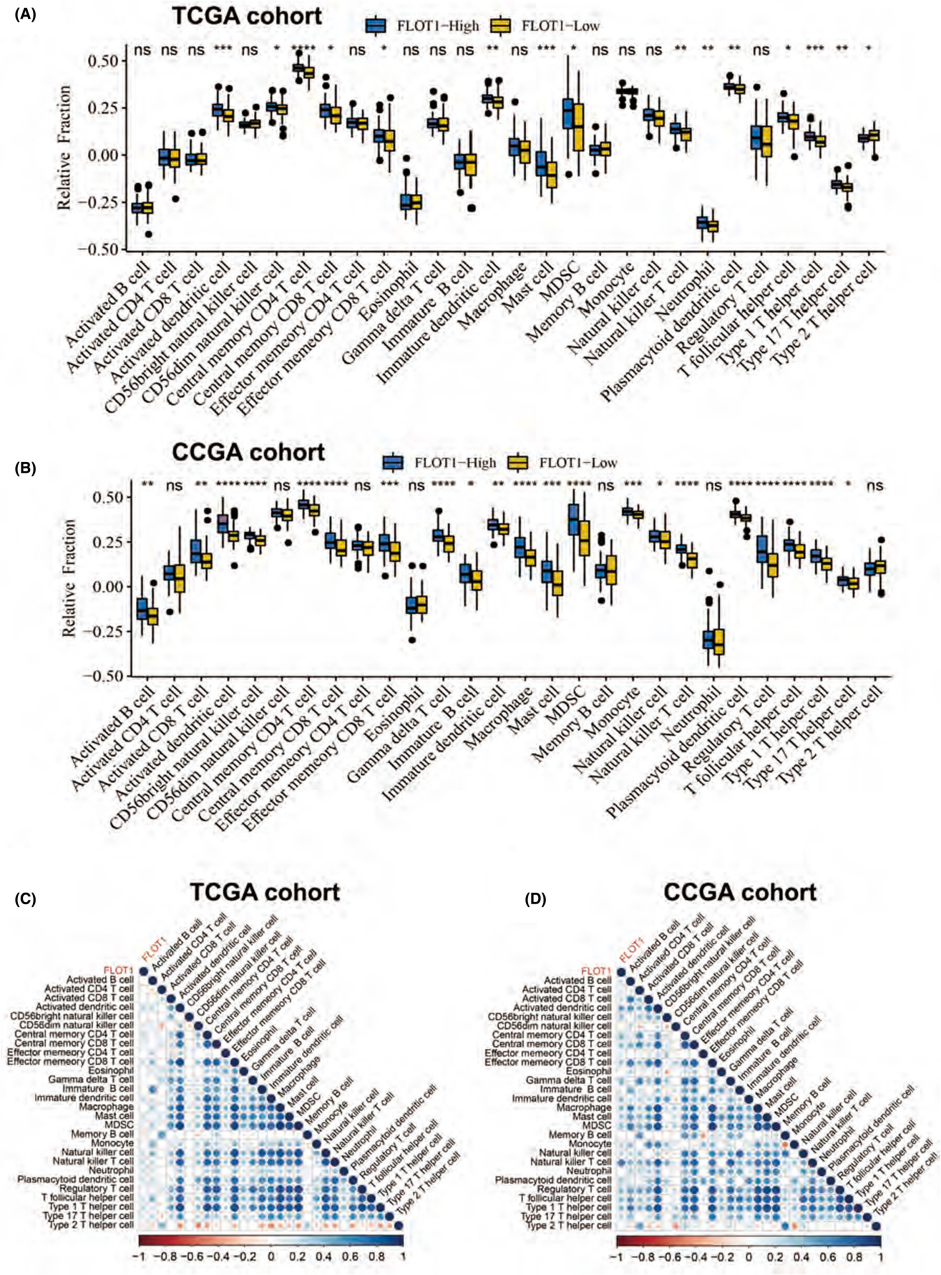
Immune infiltration of the high‐FLOT1 and the low‐FLOT1 groups. (A, B) Box plot shows comparison of immune cell fractions based on TCGA or CGGA datasets. (C, D) Heat map shows the correlation of 28 immune cell fractions based on TCGA or CGGA datasets.

According to molecular studies, infiltrating stromal and immune cells had a great impact on cancer biology and tumour signalling perturbation.^19^ Initially, we explored the association between FLOT1 expression and immune, stromal and ESTIMATES scores. According to TCGA database, the results showed a positive correlation between FLOT1 expression and immune, stromal and ESTIMATES scores (Figure 6A). Results from the CGGA database showed that FLOT1 expression was significantly associated with immune, stromal and ESTIMATES scores (Figure 6B). We also examined the types of immune cells associated with FLOT1 expression. First, according to the median value of FLOT1 expression, we divided samples into high and low groups. By using single‐sample gene set enrichment analysis, the relative abundance of 28 previously reported immune cells was quantified.^20^ Both TCGA and CGGA databases showed that FLOT1 expression was positively associated with many types of immune cells (Figure 7A–B). For TCGA cohort, we also explored the correlation between FLOT1 expression and the abundance of six major immune cell infiltrates using TIMER (Figure S2). The results were consistent with the single‐sample gene set enrichment analysis. These correlations were more significant in the CGGA database. In addition, 12 types of immune cells were significantly positively correlated with FLOT1 expression in both TCGA and CGGA databases, activated dendritic cells, central memory CD4 T cells, central memory CD8 T cells, effector memory CD8 T cells, immature dendritic cells, mast cells, myeloid‐derived suppressor cells, natural killer T cells, plasmacytoid dendritic cells, T follicular helper cells, Type 1 T helper T cells and Type 17 T helper cells (Figure 7C–D).

## 4 DISCUSSION

Although recent studies have helped understand the biological features of glioma, further investigation is required to explore the specific molecular mechanisms and therapeutic targets of this disease. In this study, we first reported FLOT1 as a potential molecular marker in the development of glioma, as evidence suggested that FLOT1 is closely associated with clinicopathological features and immune surveillance of gliomas and promoted glioma cell proliferation, invasion and migration.

FLOT1 is a molecular marker of lipid rafts, which serves as a physical platform for assembling functional complexes. Therefore, FLOT1 is involved in many biological processes, including endocytosis, adhesion, actin cytoskeleton reorganization and cell‐signalling events.^10^, ^11^, ^12^, ^13^ In addition, FLOT1 has been reported to be upregulated in many tumour types^14^, ^15^, ^16^ and is involved in cancer biology by initiating receptor kinase signalling.^23^ In this study, we found that FLOT1 is markedly upregulated in glioma tissue, compared with normal brain tissues. Moreover, according to the data from TCGA and CGGA databases, we documented that the level of FLOT1 expression was negatively correlated with the aggressiveness types and IDH status of glioma and overall survival of glioma patients, which indicated that FLOT1 was a prognostic marker for glioma patients.

Furthermore, in line with previous studies, we observed high expression level of FLOT1 in glioma cell lines and the contribution of FLOT1 in the biological function in vitro, since overexpression and silencing of FLOT1 significantly promoted and inhibited proliferation, migration and invasion, respectively. To elucidate the mechanism underlying the role of FLOT1 in glioma, we performed GO enrichment and KEGG pathway analyses. The KEGG results showed that FLOT1 is associated with regulating the actin cytoskeleton and proteasome, which is consistent with a previous study. The GO results indicated that FLOT1 might be related to transcriptional regulation and cell morphology, which may provide a basis for further exploration of the molecular mechanism.

FLOT1 has been reported to be a very important regulator of classical MAP kinase signalling. Monia Amaddii and colleague^22^ demonstrated that FLOT1 binds with three tiers of MAPK signalling simultaneously and modulates the activation of ERK directly. On the contrary, FLOT1 was also reported to be a downstream target of ERK signalling. Antje Banning and colleague^24^ found that the promoter activity of flotillins, both FLOT1 and FLOT2, increased in a MAPK‐dependent manner when stimulated by growth factor. With the knowledge above, we explored the relationship between FLOT1 and MAPK signalling in GBM using TCGA and CCGA databases and in vitro experiments. Highly consistent with previous study, our results confirmed the close relation between FLOT1 and MAPK signalling, especially ERK1/2.

Additionally, FLOT1 also showed a tight relationship with immune infiltration, as Ludwig et al.^25^ reported that Flot1−/− mice showed deficient recruitment of immune cells to inflammatory sites, since Flot1−/− neutrophils displayed reduced levels of phosphorylated myosin regulatory chain. Ficht et al.^26^ also reported both Flot1‐deficient or accumulated immune cells showed impaired migration parameters and morphological alterations. In our study, we found that FLOT1 expression was significantly associated with immune, stromal and ESTIMATES scores, particularly in the CGGA database. In addition, 12 types of immune cells were significantly positively correlated with FLOT1 expression in both the TCGA and CGGA databases. Among them are CD8+ T cells, which have long been recognized as the gold standard for antitumour immunity,^27^, ^28^, ^29^ myeloid‐derived suppressor and immature dendritic cells, both of which played important immunosuppressive roles in the process of tumour progression.^30^ Based on these findings, FLOT1 may play a potential role in the tumour microenvironment. However, the specific underlying mechanism requires further investigation.

Currently, there is no inhibitor targeting FLOT1. However, aside from GBM, FLOT1 was reported to play a role in neurological disorders, for example Alzheimer's disease,^31^ Parkinson,^32^ multiple sclerosis and biological ageing.^33^ Therefore, FLOT1 may be a potential target for brain‐related disease including tumour.

## AUTHOR CONTRIBUTIONS

**Ran Wang:** Conceptualization (equal); investigation (lead); methodology (equal); resources (equal); validation (lead); visualization (equal); writing – original draft (lead); writing – review and editing (equal). **Zhikang Chen:** Conceptualization (equal); funding acquisition (equal); project administration (equal); supervision (equal); writing – review and editing (equal). **Yi Zhang:** Data curation (equal); funding acquisition (equal); resources (equal); writing – review and editing (equal). **Shihan Xiao:** Data curation (equal); formal analysis (equal); resources (equal); writing – review and editing (equal). **Wuming Zhang:** Data curation (equal); formal analysis (equal); resources (equal); writing – review and editing (equal). **Xianqin Hu:** Data curation (equal); formal analysis (equal); resources (equal); writing – review and editing (equal). **Qun Xiao:** Data curation (equal); methodology (equal); resources (equal); software (equal); visualization (equal); writing – review and editing (equal). **Qing Liu:** Conceptualization (equal); funding acquisition (equal); project administration (equal); supervision (equal); writing – review and editing (equal). **Xiang‐Yu Wang:** Conceptualization (equal); methodology (equal); project administration (equal); resources (equal); writing – review and editing (equal).

## CONFLICT OF INTEREST

The authors declare no competing financial interests or conflicts concerning the work described.

## Supporting information


FigureS1

FigureS2

FigureS3

TableS1

TablesS2‐S6

## Data Availability

The data that support the findings of this study are available from the corresponding author upon reasonable request.
