# Supplementary figures and images for: Flotillin‐1 is a prognostic biomarker for glioblastoma and promotes cancer development through enhancing invasion and altering tumour microenvironment
Source: J Cell Mol Med. 2023 Jan 17;27(3):392–402. doi: 10.1111/jcmm.17660 (PMC9889621; doi:10.1111/jcmm.17660)

**A**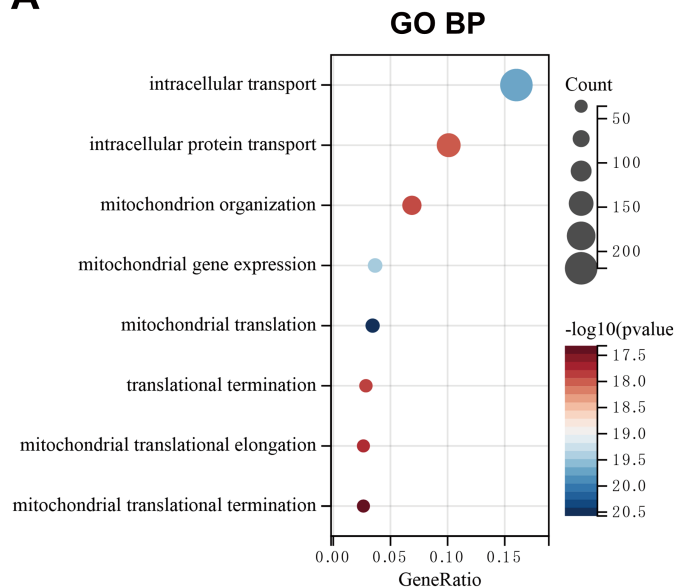**B**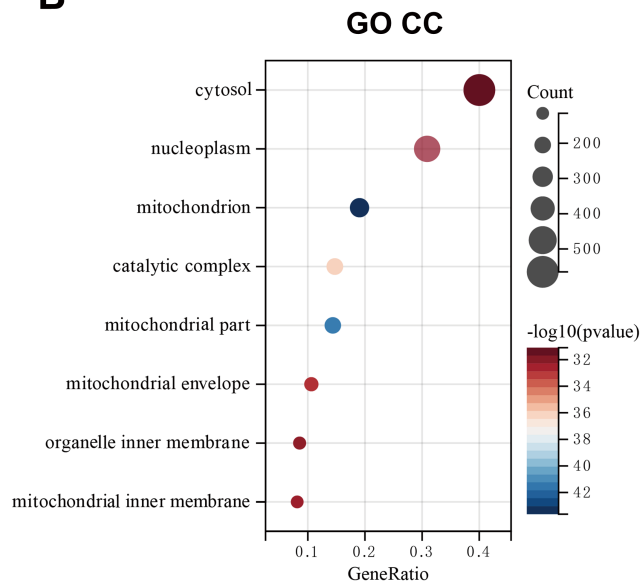**C**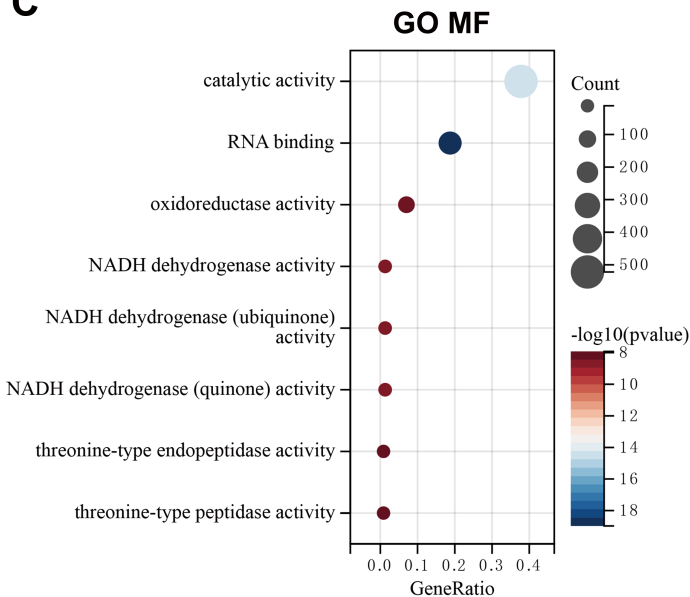**D**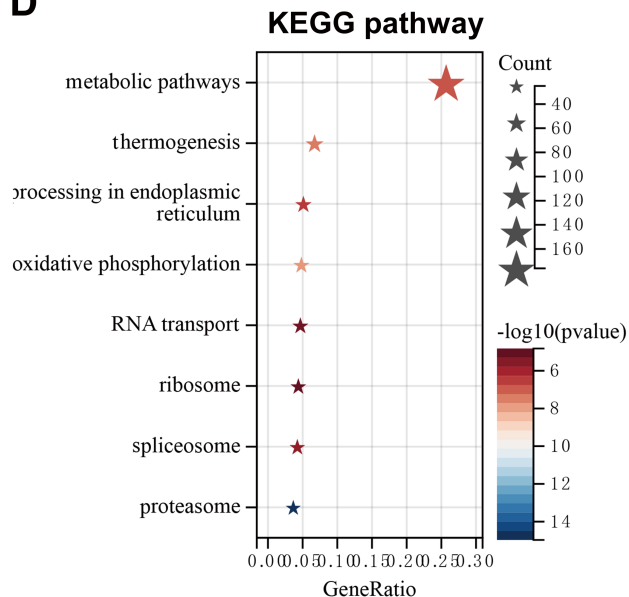

Supplement: Supplementary file 1 — FigureS1 [file JCMM-27-392-s005.pdf]

FLOT1 Expression Level(log2 TPM)

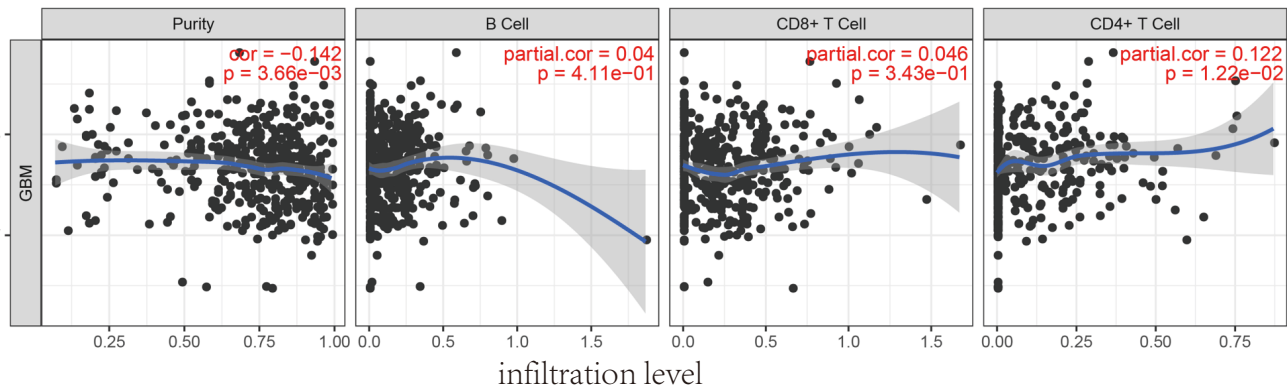

FLOT1 Expression Level(log2 TPM)

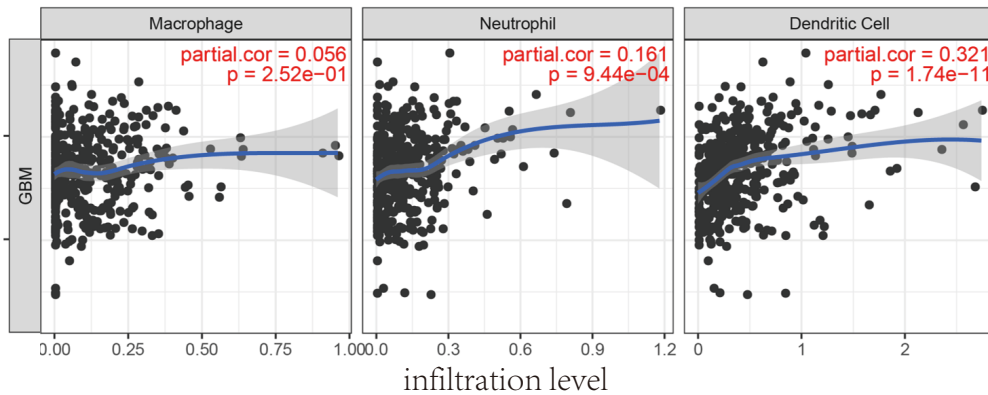

Supplement: Supplementary file 2 — FigureS2 [file JCMM-27-392-s003.pdf]

A

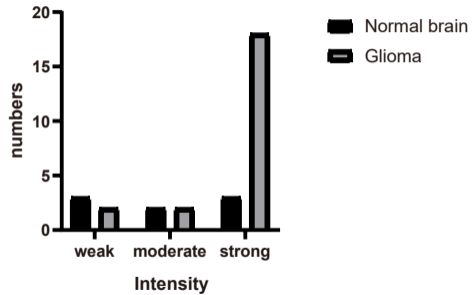

B

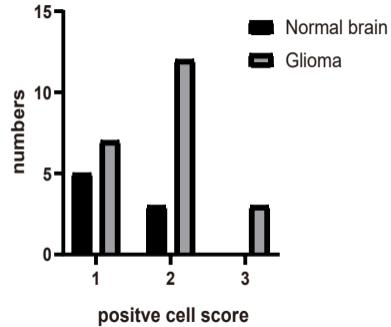

Supplement: Supplementary file 3 — FigureS3 [file JCMM-27-392-s001.pdf]
