# Supplementary material for: Flotillin‐1 is a prognostic biomarker for glioblastoma and promotes cancer development through enhancing invasion and altering tumour microenvironment
Source: J Cell Mol Med. 2023 Jan 17;27(3):392–402. doi: 10.1111/jcmm.17660 (PMC9889621; doi:10.1111/jcmm.17660)
Supplement: Supplementary file 4 — TableS1 [file JCMM-27-392-s002.docx]

Table S1. Clinical characteristics of GBM patients in the TCGA cohort and CGGA cohort.

| **Characteristics** | **TCGA cohort (N=143)** | **CGGA cohort (N=133)** |
| --- | --- | --- |
| **Age** |  |  |
| ≤60 years | 77 (53.85%) | 96 (72.18%) |
| >60 years | 66 (46.15%) | 37 (27.82%) |
| **Gender** |  |  |
| Male | 94 (65.73%) | 80 (60.15%) |
| Female | 49 (34.27%) | 53 (39.85%) |
| **IDH1 status** |  |  |
| Mutant | 6 (4.20%) | 21 (15.80%) |
| Wild-type | 133 (93.00%) | 105 (78.94%) |
| NA | 4 (2.80%) | 7 (5.26%) |
| **MGMT status** |  |  |
| Methylated | 49 (34.27%) | 63 (47.37%) |
| Unmethylated | 62 (43.36%) | 54 (40.60%) |
| NA | 32 (22.37%) | 16 (16.03%) |
| **Vital status** |  |  |
| Alive | 50 (34.97%) | 23 (17.3%) |
| Dead | 93 (65.03%) | 110 (82.7%) |
| **Subtype** |  |  |
| Classical | 55 (38.46%) | 32 (24.06%) |
| Mesenchymal | 48 (33.57%) | 31 (23.30%) |
| Proneural | 40 (27.97%) | 42 (31.57%) |
| NA | 0 | 28 (21.07%) |

NA, no data.
